# Safe provision of systemic anti-cancer treatment for urological cancer patients during COVID-19: a tertiary centre experience in the first wave of COVID-19

**DOI:** 10.1186/s12894-022-01023-6

**Published:** 2022-04-29

**Authors:** Alfred Chung Pui So, Christina Karampera, Muhammad Khan, Beth Russell, Charlotte Moss, Maria J. Monroy-Iglesias, Kiruthikah Thillai, Debra Hannah Josephs, Elias Pintus, Sarah Rudman, Mieke Van Hemelrijck, Saoirse Dolly, Deborah Enting

**Affiliations:** 1 Department of Oncology, Guy’s and St Thomas’ NHS Foundation Trust, London, SE1 9RT UK; 2 Translational Oncology and Urology Research (TOUR), School of Cancer and Pharmaceutical Sciences, King’s College London, London, SE1 9RT UK

## Abstract

**Background:**

Safe provision of systemic anti-cancer treatment (SACT) during the COVID-19 pandemic remains an ongoing concern amongst clinicians.

**Methods:**

Retrospective analysis on uro-oncology patients who continued or started SACT between 1st March and 31st May 2020 during the pandemic (with 2019 as a comparator).

**Results:**

441 patients received SACT in 2020 (292 prostate, 101 renal, 38 urothelial, 10 testicular) compared to 518 patients in 2019 (340 prostate, 121 renal, 42 urothelial, 15 testicular). In 2020, there were 75.00% fewer patients with stage 3 cancers receiving SACT (*p* < 0.0001) and 94.44% fewer patients receiving radical treatment (*p* = 0.00194). The number of patients started on a new line of SACT was similar between both years (118 in 2019 vs 102 in 2020; *p* = 0.898) but with 53.45% fewer patients started on chemotherapy in 2020 (*p* < 0.001). Overall, 5 patients tested positive for COVID-19 (one asymptomatic, one mild, two moderate, one severe resulting in death). Compared to 2019, 30-day mortality was similar (1.69% in 2019 vs 0.98% in 2020; *p* = 0.649) whereas 6-month mortality was lower (9.32% in 2019 vs 1.96% in 2020; *p* = 0.0209) in 2020.

**Conclusion:**

This study suggests that delivery of SACT to uro-oncology patients during COVID-19 pandemic may be safe in high-incidence areas with appropriate risk-reduction strategies.

**Supplementary Information:**

The online version contains supplementary material available at 10.1186/s12894-022-01023-6.

## Background

On the 23rd of March 2020, the UK government officially announced the first national lockdown in response to the COVID-19 pandemic. Over the past two years, we have seen dramatic changes in healthcare service provision, repurposing of drugs for severe COVID-19 infections, and the arrival of COVID-19 vaccines. Coming out of the third wave with anticipation of emerging new variants of COVID-19, we need to address ongoing concerns on how to deliver essential non-COVID-19 services to the public.

All aspects of cancer services have been significantly impacted by the pandemic, resulting in a backlog of patients who may present with more aggressive disease or in extremis. During the early stages of the pandemic, initial observational studies suggested that having active cancer and receiving systemic anti-cancer treatments (SACT) increased a patient’s risk of severe COVID-19 and subsequent death [1–3]. Since then, there has been significant increase in published material evaluating this risk in patients with cancer. Although the data remains heterogenous between studies due to variations in oncological characteristics, comorbidities, and study design, common risk factors for poor outcomes from COVID-19 include active cancer (especially haematological malignancies) and those similar to the general population (e.g. older adults, male sex, cardiovascular comorbidities) [4–11]. In addition, receiving SACT does not appear to be a risk factor for severe COVID-19 and death [6–12].

Whilst the national institute for health and care excellence (NICE) and international experts have provided recommendations on the prioritisation of cancer treatments, the ability to deliver these will vary depending on resources available to the healthcare provider. There have been several published articles providing practical recommendations on the management of patients with cancer during the COVID-19 pandemic [13, 14]. Our tertiary cancer centre in South London treats around 8,800 patients per annum (of which 4,500 are new patients) and is one of the largest comprehensive cancer centres in the UK. Our institute was also at the epicentre of the COVID-19 pandemic with London having the highest rates of infection and COVID-19-related deaths. Here, we report our experience in delivering SACT to patients with urological cancers during the first wave in order to support clinicians in developing guidelines for managing these patients throughout the pandemic.

## Methods

### Restructuring cancer services at Guy’s Cancer Centre

During the first wave of COVID-19 there were several key changes in how we restructured our cancer services in order to provide essential cancer services whilst minimising the risk of COVID-19 (Table 1).

**Table 1:** Strategies to reduce risk of COVID-19 transmission at Guy’s Cancer Centre

Variable	Strategy
Limiting transmission risk	*Testing patients receiving SACT at the cancer centre* Staff members were deployed to the front of hospital to ask COVID-19 screening questions and check patient temperatures prior to entering Patients who tested positive for COVID-19 but required an in-person review or were suitable to continue SACT followed a specific COVID-19 pathway within the Cancer Centre (separated from other patients). Patients receiving SACT were treated in a side room by dedicated nursing staff *Visitors* Majority of visitors and relatives were not allowed to attend the hospital with the patients. However, there were several extenuating circumstances including patients receiving end-of-life care and vulnerable individuals *Staff members* Staff members conducted basic measures to reduce risk of transmission including hand hygiene, wearing appropriate PPE depending on the clinical context, social distancing, and self-isolation if they develop symptoms suggestive of COVID-19 *Social distancing* Infection control teams helped determine the limit in which the number of people can be in a room, elevator, or waiting area
Consultations	*Virtual and telephone consultants* There has been significant increase in utilising technology to aid virtual and telephone consultations, limiting the number of potential contacts both the patient and clinician will have. This also allowed ongoing communication with the patient and their relatives during uncertain times throughout the pandemic Although this may not be appropriate for all circumstances, this was particularly useful for patients well established on treatments or at a particularly high risk of severe COVID-19 due to comorbidities *Deferring follow-up consultations* We extended the duration between follow-up consultations for some patients who were established on their current treatment. This also applied to certain routine follow-up imaging in which there was a low chance that it will impact the current treatment regimen
Outsourcing services	*Satellite hubs* These were implemented with the aid of ambulance services to provide mobile blood testing facilities. These tests were then sent to the centre and reviewed by the clinical staff This allowed patients to limit their duration of travel, avoid public transports, and limit contact with others at the cancer centre *Courier services* Patients that are established on SACT can have their medications couriered to their home rather than pick it up at the cancer centre
Treatment prioritisation	With recommendations from NICE and expert consensus, treatments were prioritised based on risk–benefit to contracting COVID-19, probability of cure, reducing immunosuppressive states, and availability of resources to deliver these services. These decisions were discussed with the patient and were considered on a case-by-case scenario

### Data analysis

All patients at a tertiary cancer centre in London receiving at least one SACT cycle for a urological cancer between 1st March 2020 and 31st May 2020 were included for data analysis. We used the same timeframe in 2019 as a comparator group. Patient demographics, oncological characteristics, SACT information, and COVID-19 status were extracted using our patient electronic records and chemotherapy prescribing system. Socioeconomic status was categorised into low, middle, and high using the English Indices of Multiple Deprivation (IMD) ranking based on postcodes. Cancers were staged according to the UICC 8th staging system. COVID-19 infection was defined as a positive RT-PCR test. Patients with only radiological changes or symptoms suggestive of COVID-19 without a positive RT-PCR test were excluded. COVID-19 positive patients were then categorised into asymptomatic, mild, moderate, or severe disease as defined by the WHO criteria [15]. Chi-square testing was used to compare demographics and clinical characteristics in 2020 with 2019. A *p*-value of < 0.05 was considered statistically significant. Subsequent post-hoc subgroup analysis based on a statistically significant Chi-square test was performed using the Bonferroni correction for multiple comparisons. All data was collected as part of Guy’s Cancer Cohort (Ethics Reference number: 18/NW/0297) [16].

## Results

In the study period, there was a total of 441 patients who received SACT in 2020 (292 prostate, 101 renal, 38 urothelial, 10 testicular) compared to 518 patients in 2019 (340 prostate, 121 renal, 42 urothelial, 15 testicular) with an overall decline of 14.86% (Table 2). Overall, there was a reduction in the number of patients receiving SACT in 2020 during the first wave of the COVID-19 pandemic with the largest reductions seen in prostate (14.12%) and renal cancers (16.53%). There were no significant differences in patient demographics with regards to age, sex, socioeconomic status, and ethnicity (Table 3, Additional file 1: Table S1). There was a significant amount of missing data on patient performance status in 2020 (26.35%; *p* < 0.0001) which made it difficult to comment on any differences.

**Table 2:** Oncological characteristics of patients receiving SACT

	2019 (*n* = 518)		2020 (*n* = 441)		(*n*_2020_–*n*_2019_)/*n*_2019_ (%)	*p* value
	*n*	%	*N*	%		
Cancer type						
Prostate	340	65.64	292	66.21	14.12	0.851
Renal	121	23.36	101	22.90	16.53	0.867
Urothelial	42	8.11	38	8.62	9.52	0.776
Testicular	15	2.90	10	2.27	33.33	0.543
Stage						
1	7	1.35	2	0.45	71.43	0.161
2	11	2.12	6	1.36	45.45	0.368
3	36	6.95	9	2.04	75.00	< 0.0001*
4	463	89.38	423	95.92	8.64	< 0.0001*
Missing	1	0.19	1	0.23	0.00	0.271
SACT						
Chemotherapy	112	21.62	71	16.10	36.61	0.0278
Immunotherapy (IO)	43	8.30	50	11.34	− 16.28	0.110
Hormone	244	47.10	236	53.51	3.28	0.0455
Biological/targeted	102	19.69	67	15.19	34.31	0.0574
Combo (Chemo/hormone)	9	1.74	2	0.45	77.78	0.0574
Combo (Chemo/IO)	1	0.19	1	0.23	0.00	0.920
Combo (Chemo/target)	0	0.00	1	0.23	N.A	0.271
Combo (IO/Hormone)	1	0.19	0	0.00	100.00	0.368
Combo (IO/target)	5	0.97	13	2.95	− 160.00	0.0214
Combo (Chemo/IO/hormone)	1	0.19	0	0.00	100.00	0.368
Treatment paradigm						
Neoadjuvant	11	2.12	6	1.36	45.45	0.368
Adjuvant	17	3.28	6	1.36	64.71	0.0574
Radical	18	3.47	1	0.23	94.44	0.00194*
Palliative	470	90.73	419	95.01	10.85	0.0124
Curative	2	0.39	9	2.04	− 350.00	0.0164
Line of palliative treatment (2019, n = 470; 2020, n = 419)						
1	94	20.00	117	27.92	− 24.47	0.00194*
2	248	52.77	257	61.34	− 3.63	0.00137*
3	87	18.51	30	7.16	65.52	< 0.0001*
4	26	5.53	10	2.39	61.54	0.0278
5	11	2.34	3	0.72	72.73	0.0574
6	2	0.43	2	0.48	0.00	0.841
7	1	0.21	0	0.00	100.00	0.368
Missing	1	0.21	0	0.00	100.00	0.368
Trial treatment						
Yes	64	12.36	52	11.79	18.75	0.790
SACT initiated during study period						
Yes	118	22.78	102	23.13	13.56	0.898

*Statistically significant *p* values after Bonferroni correction for multiple comparisons

**Table 3:** Patient demographics

	2019 (*n* = 518)		2020 (*n* = 441)		(*n*_2020_–*n*_2019_)/*n*_2019_ (%)	*p* value
	*n*	%	*n*	%		
Sex						
Male	467	90.15	402	91.16	13.92	0.596
Female	51	9.85	39	8.84	23.53	0.596
Age						
< 50	26	5.02	24	5.44	7.69	0.769
50–59	62	11.97	49	11.11	20.97	0.679
60–69	164	31.66	152	34.47	7.32	0.357
70–79	178	34.36	139	31.52	21.91	0.351
≥ 80	88	16.99	77	17.46	12.50	0.847
Mean (SD)—years	68.8 (11.5)	–	68.6 (11.3)	–	–	
Socioeconomic status (IMD)						
Low	168	32.43	145	32.88	13.69	0.883
Middle	170	32.82	143	32.43	15.88	0.897
High	178	34.36	152	34.47	14.61	0.973
Missing	2	0.39	1	0.23	50.00	0.660
Ethnicity						
White British	239	46.14	190	43.08	20.50	0.343
White Other	26	5.02	28	6.35	− 7.69	0.373
Black Caribbean	36	6.95	21	4.76	41.67	0.153
Black African	17	3.28	19	4.31	− 11.76	0.405
Black Other	4	0.77	5	1.13	− 25.00	0.563
Asian	13	2.51	7	1.59	46.15	0.319
Mixed	1	0.19	0	0.00	100.00	0.356
Other	8	1.54	7	1.59	12.50	0.957
Unknown	174	33.59	164	37.19	5.75	0.245
Performance status (ECOG)						
0	138	26.64	102	23.13	26.09	0.194
1	323	62.36	204	46.26	36.84	< 0.0001*
2	49	9.46	16	3.63	67.35	< 0.001*
3	8	1.54	2	0.45	75.00	0.0357
Missing	0	0.00	117	26.53	N.A	< 0.0001*

*Statistically significant *p* values after Bonferroni correction for multiple comparisons

The majority of the patients had advanced or metastatic cancers (stage 3–4). There was a greater decline in the proportion of patients who received SACT with stage 3 cancers (75.00%; *p* < 0.0001) compared to stage 4 cancers (8.42%; *p* < 0.0001) in 2020. This difference was best observed with prostate cancer where there were 18 fewer patients with stage 3 cancers who received SACT in 2020 (20 vs 2; *p* < 0.001) (Additional file 1: Table S2).

Hormone treatment was the most common type of SACT delivered followed by chemotherapy, targeted therapy, and immunotherapy in both 2019 and 2020. The largest reductions were seen with chemotherapy (36.61%; *p* = 0.0278) and targeted therapy (34.31%; *p* = 0.0574). This was particularly evident in renal cancers with a decline of 42.71% in the number of patients receiving targeted therapy (*p* < 0.0001) (Additional file 1: Table S2). Furthermore, there was a small increase in the number of patients with renal cancer receiving immunotherapy alone and in combination with targeted treatment. In the prostate cancer group, whilst there was a reduction in number of patients receiving chemotherapy in 2020 (32.91%; *p* = 0.00689), there was no significant difference in the number of patients receiving hormone therapy (Additional file 1: Table S2). The majority of the prostate cancer group receiving hormone therapy were treated with novel hormone agents (i.e. abiraterone, enzalutamide) in both 2019 (94.26%) and 2020 (91.95%) (Additional file 1: Table S2). Unfortunately, due to the pandemic there were generally fewer urological patients receiving SACT as part of a radical regimen (from 3.47 to 0.23%; *p* = 0.00194) and fewer patients going onto 3rd line palliative SACT (from 16.80 to 6.80%; *p* < 0.0001) (Table 2). The number of patients on clinical trial treatments were similar between 2019 and 2020 (64 vs 52; *p* = 0.790).

The number of patients that were started on a new line of SACT was similar between both years (118 vs 102; *p* = 0.898). However, further subgroup analysis suggests that there were less patients with prostate cancer being started on SACT (74 vs 57; *p* = 0.488) and less patients with urological cancers started on chemotherapy (58 vs 27; *p* < 0.001) in 2020 (Table 4). The type of patients that were started on SACT during COVID-19 were generally younger with a performance status between 0 and 1. The majority received palliative SACT and had similar number of lines of palliative treatment. Fewer patients with stage 1 disease (primarily testicular cancers) were started on SACT and fewer patients received adjuvant SACT. The number of patients starting on curative or radical treatments were similar between both years. In patients started on SACT during COVID-19, the 30-day mortality was similar (1.69% vs 0.98%; *p* = 0.649) compared with 2019 (Table 4). On the other hand, the 6-month mortality was lower in 2020 (9.32% vs 1.96%; *p* = 0.0209).

**Table 4:** Patient demographics and oncological characteristics of patients started on SACT between 1st March to 31st May in 2020 during COVID-19 (with 2019 as a comparator)

	2019 (*n* = 118)			2020 (*n* = 102)			(*n*_2020_–*n*_2019_)/*n*_2019_ (%)	*p* value
	*n*	%		*n*		%		
Sex								*N*
Male	106	89.83		90		88.24	15.09	0.705
Age								
Mean (SD)—years	66.92 (12.16)	–		65.12 (13.23)		–	–	–
Socioeconomic status (IMD)								
Low	37	31.36		40		39.22	− 8.11	0.223
Middle	43	36.44		28		27.45	34.88	0.155
High	38	32.20		33		32.35	13.16	0.981
Missing	0	0.00		1		0.98	N.A	0.281
Ethnicity								
White British	44	37.29		29		28.43	34.09	0.164
White Other	9	7.63		9		8.82	0.00	0.747
Black Caribbean	9	7.63		5		4.90	44.44	0.409
Black African	3	2.54		4		3.92	− 33.33	0.561
Black Other	0	0.00		1		0.98	N.A	0.281
Asian	1	0.85		0		0.00	100.00	0.351
Mixed	1	0.85		0		0.00	100.00	0.351
Other	1	0.85		1		0.98	0.00	0.917
Unknown	50	42.37		53		51.96	− 6.00	0.155
Performance status (ECOG)								
0	42	35.59		27		26.47	35.71	0.134
1	62	52.54		61		59.80	1.61	0.271
2	13	11.02		3		2.94	76.92	0.0214
3	1	0.85		0		0.00	100.00	0.368
Missing	0	0.00		11		10.78	N.A	0.000216*
Cancer type								
Prostate	74		62.71		57	55.88	22.97	0.303
Renal	22		18.64		21	20.59	4.55	0.717
Urothelial	14		11.86		17	16.67	− 21.43	0.307
Testicular	8		6.78		7	6.86	12.50	0.981
Stage								
1	7		5.93		1	0.98	85.71	0.0504
2	4		3.39		5	4.90	− 25.00	0.572
3	7		5.93		4	3.92	42.86	0.495
4	100		84.75		92	90.20	8.00	0.226
Missing	0		0.00		0	0.00	N.A	–
SACT								
Chemotherapy	58		49.15		27	26.47	53.45	0.000674*
Immunotherapy	15		12.71		18	17.65	− 20.00	0.230
PD-1/L1	11		9.32		15	14.71	− 36.36	–
PD-1/L1 + CTLA-4	3		2.54		4	3.92	− 33.33	–
Vaccine	1		0.85		0	0.00	100.00	–
Hormone	33		27.97		43	42.16	− 30.30	0.0278
Novel hormone agents	33		27.97		41	40.20	− 24.24	–
Biological/targeted	11		9.32		12	11.76	− 9.09	0.689
Combo (Chemo/hormone)	1		0.85		1	0.98	0.00	0.920
Combo (IO/target)	0		0.00		1	0.98	N.A	0.271
Treatment paradigm								
Neoadjuvant	8		6.78		4	3.92	50.00	0.368
Adjuvant	10		8.47		2	1.96	80.00	0.0357
Radical	4		3.39		0	0.00	100.00	0.0574
Palliative	95		80.51		90	88.24	5.26	0.110
Curative	1		0.85		6	5.88	− 500.00	0.0357
Line of palliative treatment (2019, n = 95; 2020, n = 90)								
1	24		20.34		28	27.45	− 16.67	0.376
2	49		41.53		50	49.02	− 2.04	0.588
3	14		11.86		8	7.84	42.86	0.219
4	5		4.24		2	1.96	60.00	0.279
5	2		1.69		1	0.98	50.00	0.593
6	0		0.00		1	0.98	N.A	0.303
7	1		0.85		0	0.00	100.00	0.329
Trial treatment								
Yes	7		5.93		3	2.94	57.14	0.288
30-day mortality	2	1.69		1		0.98	50.00	0.649
6-month mortality	11	9.32		2		1.96	81.82	0.0209*

*Statistically significant *p* values after Bonferroni correction for multiple comparisons

Of the 441 patients who received SACT during the study period, 5 tested positive for COVID-19 (2 prostate, 2 renal, 1 bladder) (Table 5). All patients were male, ≥ 60 years of age, had stage 4 urological cancer and receiving palliative SACT (2 hormone, 2 targeted, 1 immunotherapy). In addition, 4 were from a lower socioeconomic background, 3 had more than one comorbidity, and 3 had polypharmacy. With regards to COVID-19 severity, 1 patient had asymptomatic infection, 1 had mild infection, 2 had moderate COVID-19 pneumonitis, and 1 died from COVID-19. The patient who died from severe COVID-19 pneumonitis with thromboembolic complications had metastatic bladder cancer and was receiving palliative targeted therapy.

**Table 5:** Patient demographics and oncological characteristics of patients tested positive for COVID-19

	2020 (*n* = 5)	
	*n*	%
*Patient demographics*		
Sex		
Male	5	100.00
Age		
Mean (SD)—years	60.4 (12.9)	
Socioeconomic status (IMD)		
Low	4	80.00
Missing	1	20.00
Ethnicity		
White British	2	40.00
Black African	1	20.00
Other	1	20.00
Unknown	1	20.00
*Associated comorbidities*		
Comorbidities		
Hypertension	3	60.00
Diabetes	3	60.00
Lung conditions	0	0.00
Renal impairment	1	20.00
Liver conditions	0	0.00
Cerebrovascular disease	0	0.00
Frailty	1	20.00
Long-term steroid use	0	0.00
Number of comorbidities		
0	2	40.00
1	0	0.00
2	1	20.00
3 or more	2	40.00
Medications		
Polypharmacy	3	60.00
NSAIDs	0	0.00
ACE/ARB	0	0.00
Beta-blockers	0	0.00
*Oncological characteristics*		
Cancer type		
Prostate	2	40.00
Renal	2	40.00
Bladder	1	20.00
Testicular	0	0.00
SACT		
Chemotherapy	0	0.00
Immunotherapy	1	20.00
Biological/targeted	2	40.00
Hormone	2	40.00
Treatment paradigm		
Palliative	5	100.00
*COVID-19 severity*		
COVID-19 severity (WHO criteria)		
Asymptomatic	1	20.00
Mild	1	20.00
Moderate pneumonia	2	40.00
Severe pneumonia	0	0.00
COVID-related death	1	20.00

## Discussion

In this single-centre retrospective cohort study, we report the outcomes of urological oncology patients receiving SACT during the first wave of the COVID-19 pandemic. Despite an inevitable decline in the number of patients receiving SACT during COVID-19, we were able to provide a safe high-quality urological cancer SACT pathway with a low incidence of COVID-19 infections (0.73%). The low rates of COVID-19 infections in our patients during the first wave would have also been impacted by national lockdown procedures and high levels of shielding due to our clinically vulnerable groups. Of the urological cancers, patients with prostate and renal cancers were most affected with reductions in delivery of both chemotherapy and targeted therapy respectively. This reflects clinical decision making whereby on a case-by-case basis treatment was either deferred or commenced without delay based on symptoms, growth rate of cancer, and patient factors. For instance, patients with slow growing metastatic renal cancers might have been advised to delay starting SACT for a short number of months in order to avoid hospital visits, risk of infection, and toxicities. Whereas patients diagnosed with advanced urothelial carcinoma were generally recommended to start SACT since a delay would likely lead to progressive disease over a short time period. Although there was a small increase in the number of patients receiving combination targeted/immunotherapy, this reflects the availability of axitinib/avelumab for untreated advanced renal cancers rather than an effect of the pandemic [17]. Reassuringly, the number of patients initiated on a new line of SACT was similar during COVID-19, albeit with fewer patients started on chemotherapy.

These differences reflect initial concerns regarding chemotherapy as a potential risk factor for severe COVID-19 [1–3]. However, there is an increasing body of evidence that challenges this notion [6–12]. The current evidence does not suggest SACT as a risk factor for COVID-19, with the exception of haematological malignancies, and current clinical practice should therefore reflect this [6–12]. Although treatment prioritisation may explain the numbers of patients initiated on SACT, it is important to review other potential confounders. A major concern with cancer services during the pandemic is the decline in patients with cancer-related symptoms seeking medical attention during COVID-19. The number of GP appointments, 2-week waits, and core cancer diagnostic services were all significantly reduced during the first wave [18]. We are only starting to see some recovery in these statistics. Recent modelling studies and real-world data have suggested an increase in ‘missed’ cancers and shift towards higher staging [19–21]. Some of these patients may require SACT and therefore may partly explain the findings of our study. Another factor to consider is the availability of resources and personnel to deliver SACT due to redeployment to emergency and critical care services during COVID-19.

The decline in 6-month all-cause mortality during COVID-19 was another interesting finding. However, this figure does not take into account the proportion of patients who died from cancer and did not receive SACT due to the potential risk of contracting COVID-19 outweighing any survival benefits. There may also be an element of selection bias as the patients who were receiving SACT during COVID-19 were generally younger and of good performance status. There may also be a general decline in deaths from other nosocomial and community infectious diseases due to shielding, social distancing, and increased vigilance in infection control protocols [22–25].

It is important to appreciate the certain limitations to our study. The main limitation was that we only included patients who received SACT during COVID-19 and did not include all patients who were potentially eligible for SACT but were not given it either due to patient decision or as a result of treatment prioritisation with risk of COVID-19 infection. Therefore, we cannot comment on mortality outcomes due to this. We also only included patients with a positive COVID-19 RT-PCR test. This would have likely missed patients who were self-isolating with mild symptoms who did not get tested, death certificate diagnosis of COVID-19 in patients presenting in extremis without time for an RT-PCR test, and false negative RT-PCR results. Another limitation is the proportion of patients with incomplete data on ethnicity and performance status in the patient electronic records. This is particularly relevant as there is growing evidence that there are significant racial and socioeconomic disparities in healthcare access during COVID-19. The method of data extraction from our electronic chemotherapy prescribing system also underrepresents the number of patients with prostate cancer receiving anti-gonadotropic monotherapy (e.g. LHRH analgoues) as the prescriptions are continued by GPs. Finally, we do not have data regarding the provision of SACT during the second wave of COVID-19. This would be an important aspect for us to study as there were more variables to consider including availability of COVID-19 treatments, emerging safety evidence of SACT, expert consensus statements, and the arrival of novel vaccines.

## Conclusion

This single-centre retrospective study demonstrated that patients could receive a range of SACT during COVID-19 with a low incidence of infection rate and mortality. Although shifts in the type of SACT delivered were observed with less chemotherapy administered, we were able to continue to start patients on SACT. With emerging new variants and easing of national lockdown measures, we hope that our data provides reassurance that SACT can be safely delivered during a pandemic with appropriate safety provisions in place. Furthermore, current strategies should also include stringent vaccination programs for patients with cancer considering the availability of COVID-19 vaccines and emerging data on its reduced efficacy in this population [26].

## Supplementary Information

**Additional file 1: Table S1.** Patient demographics of prostate, renal, urothelial, and testicular cancer groups. **Table S2.** Oncological characteristics of prostate, renal, urothelial, and testicular cancer groups.

## Data Availability

The datasets generated and analysed during the current study are not publicly available due to potential inferable data that may impact patient confidentiality. The reason being that our paper specifically refers to our institution’s practices during the pandemic within a specific timeframe. Anonymising the data may impact interpretation of the analysis but is available from the corresponding author on reasonable request.
