# Supplementary material for: Safe provision of systemic anti-cancer treatment for urological cancer patients during COVID-19: a tertiary centre experience in the first wave of COVID-19
Source: BMC Urol. 2022 Apr 29;22:71. doi: 10.1186/s12894-022-01023-6 (PMC9051846; doi:10.1186/s12894-022-01023-6)
Supplement: Supplementary file 1 — Additional file 1: Table S1. Patient demographics of prostate, renal, urothelial, and testicular cancer groups. Table S2. Oncological characteristics of prostate, renal, urothelial, and testicular cancer groups. [file 12894_2022_1023_MOESM1_ESM.docx]

Table S1: Patient demographics of prostate, renal, urothelial, and testicular cancer groups.

|  | Prostate cancer | | | | | Renal cancer | | | | | Urothelial cancer | | | | | Testicular cancer | | | | |
| --- | --- | --- | --- | --- | --- | --- | --- | --- | --- | --- | --- | --- | --- | --- | --- | --- | --- | --- | --- | --- |
|  | 2019 (*n*=340) | | 2020 (*n*=292) | | *p*-value | 2019 (*n*=121) | | 2020 (*n*=101) | | *p*-value | 2019 (*n*=42) | | 2020 (*n*=38) | | *p*-value | 2019 (*n*=15) | | 2020 (*n*=10) | | *p*-value |
|  | ***n*** | **%** | ***n*** | **%** |  | ***n*** | **%** | ***n*** | **%** |  | ***n*** | **%** | ***n*** | **%** |  | ***n*** | **%** | ***n*** | **%** |  |
| Sex |  |  |  |  |  |  |  |  |  |  |  |  |  |  |  |  |  |  |  |  |
| Male | 339 | 99.71% | 292 | 100.00% | 0.354 | 84 | 69.42% | 75 | 74.26% | 0.426 | 29 | 69.05% | 25 | 65.79% | 0.756 | 15 | 100.00% | 10 | 100.00% | – |
| Female | 1 | 0.29% | 0 | 0.00% | 0.354 | 37 | 30.58% | 26 | 25.74% | 0.426 | 13 | 30.95% | 13 | 34.21% | 0.756 | 0 | 0.00% | 0 | 0.00% | – |
| Age |  |  |  |  |  |  |  |  |  |  |  |  |  |  |  |  |  |  |  |  |
| <50 | 2 | 0.59% | 4 | 1.37% | 0.312 | 10 | 8.26% | 8 | 7.92% | 0.926 | 2 | 4.76% | 2 | 5.26% | 0.918 | 12 | 80.00% | 10 | 100.00% | 0.132 |
| 50-59 | 29 | 8.53% | 25 | 8.56% | 0.988 | 24 | 19.83% | 19 | 18.81% | 0.848 | 8 | 19.05% | 5 | 13.16% | 0.476 | 1 | 6.67% | 0 | 0.00% | 0.405 |
| 60-69 | 110 | 32.35% | 103 | 35.27% | 0.439 | 40 | 33.06% | 37 | 36.63% | 0.577 | 13 | 30.95% | 12 | 31.58% | 0.952 | 1 | 6.67% | 0 | 0.00% | 0.405 |
| 70-79 | 124 | 36.47% | 94 | 32.19% | 0.259 | 38 | 31.40% | 30 | 29.70% | 0.784 | 16 | 38.10% | 15 | 39.47% | 0.899 | 0 | 0.00% | 0 | 0.00% | – |
| ≥80 | 75 | 22.06% | 66 | 22.60% | 0.870 | 9 | 7.44% | 7 | 6.93% | 0.884 | 3 | 7.14% | 4 | 10.53% | 0.593 | 1 | 6.67% | 0 | 0.00% | 0.405 |
| Mean (SD) | 71.8 (9.2) | – | 71.1 (9.4) | – | – | 65.3 (10.8) | – | 65.2 (9.9) | – | – | 66.7 (9.5) | – | 68.0 (9.4) | – | – | 38.5 (16.3) | – | 31.9 (6.6) | – | – |
| Socioeconomic status (IMD) |  |  |  |  |  |  |  |  |  |  |  |  |  |  |  |  |  |  |  |  |
| Low | 115 | 33.82% | 103 | 35.27% | 0.702 | 36 | 29.75% | 31 | 30.69% | 0.879 | 10 | 23.81% | 7 | 18.42% | 0.556 | 7 | 46.67% | 4 | 40.00% | 0.742 |
| Mid | 108 | 31.76% | 91 | 31.16% | 0.871 | 45 | 37.19% | 38 | 37.62% | 0.947 | 11 | 26.19% | 12 | 31.58% | 0.595 | 6 | 40.00% | 2 | 20.00% | 0.294 |
| High | 116 | 34.12% | 97 | 33.22% | 0.812 | 39 | 32.23% | 32 | 31.68% | 0.931 | 21 | 50.00% | 19 | 50.00% | 1 | 2 | 13.33% | 4 | 40.00% | 0.126 |
| Missing | 1 | 0.29% | 1 | 0.34% | 0.914 | 1 | 0.83% | 0 | 0.00% | 0.360 | 0 | 0.00% | 0 | 0.00% | – | 0 | 0.00% | 0 | 0.00% | – |
| Ethnicity |  |  |  |  |  |  |  |  |  |  |  |  |  |  |  |  |  |  |  |  |
| White British | 148 | 43.53% | 117 | 40.07% | 0.379 | 65 | 53.72% | 53 | 52.48% | 0.853 | 23 | 54.76% | 19 | 50.00% | 0.670 | 3 | 20.00% | 1 | 10.00% | 0.504 |
| White Other | 14 | 4.12% | 15 | 5.14% | 0.541 | 9 | 7.44% | 11 | 10.89% | 0.371 | 3 | 7.14% | 1 | 2.63% | 0.355 | 0 | 0.00% | 1 | 10.00% | 0.211 |
| Black Caribbean | 34 | 10.00% | 19 | 6.51% | 0.114 | 2 | 1.65% | 2 | 1.98% | 0.855 | 0 | 0.00% | 0 | 0.00% | – | 0 | 0.00% | 0 | 0.00% | – |
| Black African | 14 | 4.12% | 17 | 5.82% | 0.323 | 2 | 1.65% | 2 | 1.98% | 0.855 | 1 | 2.38% | 0 | 0.00% | 0.338 | 0 | 0.00% | 0 | 0.00% | – |
| Black Other | 4 | 1.18% | 5 | 1.71% | 0.571 | 0 | 0.00% | 0 | 0.00% | – | 0 | 0.00% | 0 | 0.00% | – | 0 | 0.00% | 0 | 0.00% | – |
| Asian | 6 | 1.76% | 5 | 1.71% | 0.960 | 6 | 4.96% | 2 | 1.98% | 0.236 | 0 | 0.00% | 0 | 0.00% | – | 1 | 6.67% | 0 | 0.00% | 0.405 |
| Mixed | 0 | 0.00% | 0 | 0.00% | – | 0 | 0.00% | 0 | 0.00% | – | 0 | 0.00% | 0 | 0.00% | – | 1 | 6.67% | 0 | 0.00% | 0.405 |
| Other | 7 | 2.06% | 5 | 1.71% | 0.750 | 0 | 0.00% | 1 | 0.99% | 0.273 | 0 | 0.00% | 0 | 0.00% | – | 1 | 6.67% | 1 | 10.00% | 0.763 |
| Unknown | 113 | 33.24% | 109 | 37.33% | 0.282 | 37 | 30.58% | 30 | 29.70% | 0.887 | 15 | 35.71% | 18 | 47.37% | 0.290 | 9 | 60.00% | 7 | 70.00% | 0.610 |
| Performance status (ECOG) |  |  |  |  |  |  |  |  |  |  |  |  |  |  |  |  |  |  |  |  |
| 0 | 97 | 28.53% | 69 | 23.63% | 0.163 | 15 | 12.40% | 19 | 18.81% | 0.186 | 16 | 38.10% | 7 | 18.42% | 0.0522 | 10 | 66.67% | 7 | 70.00% | 0.861 |
| 1 | 209 | 61.47% | 117 | 40.07% | <0.0001* | 87 | 71.90% | 61 | 60.40% | 0.0702 | 22 | 52.38% | 23 | 60.53% | 0.463 | 5 | 33.33% | 3 | 30.00% | 0.861 |
| 2 | 29 | 8.53% | 10 | 3.42% | 0.00784 | 17 | 14.05% | 3 | 2.97% | 0.00409* | 3 | 7.14% | 3 | 7.89% | 0.899 | 0 | 0.00% | 0 | 0.00% | – |
| 3 | 5 | 1.47% | 2 | 0.68% | 0.347 | 2 | 1.65% | 0 | 0.00% | 0.194 | 1 | 2.38% | 0 | 0.00% | 0.338 | 0 | 0.00% | 0 | 0.00% | – |
| Missing | 0 | 0.00% | 94 | 32.19% | <0.0001* | 0 | 0.00% | 18 | 17.82% | <0.0001* | 0 | 0.00% | 5 | 13.16% | 0.0152 | 0 | 0.00% | 0 | 0.00% | – |
| *Statistically significant *p*-values after Bonferroni correction for multiple comparisons | | | | | | | | | | | | | | | | | | | | |

**Table S2:** Oncological characteristics of prostate, renal, urothelial, and testicular cancer groups.

|  | Prostate cancer | | | | | Renal cancer | | | | | Urothelial cancer | | | | | Testicular cancer | | | | |
| --- | --- | --- | --- | --- | --- | --- | --- | --- | --- | --- | --- | --- | --- | --- | --- | --- | --- | --- | --- | --- |
|  | 2019 (*n*=340) | | 2020 (*n*=292) | | *p*-value | 2019 (*n*=121) | | 2020 (*n*=101) | | *p*-value | 2019 (*n*=42) | | 2020 (*n*=38) | | *p*-value | 2019 (*n*=15) | | 2020 (*n*=10) | | *p*-value |
|  | ***n*** | **%** | ***n*** | **%** |  | ***n*** | **%** | ***n*** | **%** |  | ***n*** | **%** | ***n*** | **%** |  | ***n*** | **%** | ***n*** | **%** |  |
| Stage |  |  |  |  |  |  |  |  |  |  |  |  |  |  |  |  |  |  |  |  |
| 1 | 0 | 0.00% | 1 | 0.34% | 0.280 | 0 | 0.00% | 0 | 0.00% | – | 2 | 4.76% | 1 | 2.63% | 0.616 | 5 | 33.33% | 1 | 10.00% | 0.181 |
| 2 | 2 | 0.59% | 1 | 0.34% | 0.280 | 0 | 0.00% | 0 | 0.00% | – | 3 | 7.14% | 0 | 0.00% | 0.0931 | 6 | 40.00% | 5 | 50.00% | 0.622 |
| 3 | 20 | 5.88% | 2 | 0.68% | 0.000380* | 3 | 2.48% | 0 | 0.00% | 0.111 | 9 | 21.43% | 3 | 7.89% | 0.0904 | 4 | 26.67% | 4 | 40.00% | 0.484 |
| 4 | 318 | 93.53% | 288 | 98.63% | 0.00129* | 117 | 96.69% | 101 | 100.00% | 0.0652 | 28 | 66.67% | 34 | 89.47% | 0.0147 | 0 | 0.00% | 0 | 0.00% | – |
| Missing | 0 | 0.00% | 0 | 0.00% | – | 1 | 0.83% | 0 | 0.00% | 0.360 | 0 | 0.00% | 0 | 0.00% | *–* | 0 | 0.00% | 0 | 0.00% | – |
| SACT |  |  |  |  |  |  |  |  |  |  |  |  |  |  |  |  |  |  |  |  |
| Chemotherapy | 79 | 23.24% | 43 | 14.73% | 0.00689 | 1 | 0.83% | 2 | 1.98% | 0.458 | 17 | 40.48% | 16 | 42.11% | 0.882 | 15 | 100.00% | 10 | 100.00% | – |
| Immunotherapy (IO) | 1 | 0.29% | 2 | 0.68% | 0.476 | 19 | 15.70% | 30 | 29.70% | 0.0123 | 23 | 54.76% | 18 | 47.37% | 0.509 | 0 | 0.00% | 0 | 0.00% | – |
| Hormone | 244 | 71.76% | 236 | 80.82% | 0.00791 | 0 | 0.00% | 0 | 0.00% | – | 0 | 0.00% | 0 | 0.00% | – | 0 | 0.00% | 0 | 0.00% | – |
| Novel hormone agents | 230 | 94.26% | 217 | 91.95 | 0.0662 | – | – | – | – | – | – | – | – | – | – | – | – | – | – | – |
| Apalutamide/ Bicalutamide | 10 | 4.10% | 10 | 4.24 | 0.729 | – | – | – | – | – | – | – | – | – | – | – | – | – | – | – |
| Oestrogen | 2 | 0.82% | 3 | 1.27 | 0.534 | – | – | – | – | – | – | – | – | – | – | – | – | – | – | – |
| Biological/ Targeted | 5 | 1.47% | 9 | 3.08% | 0.170 | 96 | 79.34% | 55 | 54.46% | <0.0001* | 1 | 2.38% | 3 | 7.89% | 0.258 | 0 | 0.00% | 0 | 0.00% | – |
| Combo (Chemo/ Hormone) | 9 | 2.65% | 2 | 0.68% | 0.0600 | 0 | 0.00% | 0 | 0.00% | – | 0 | 0.00% | 0 | 0.00% | – | 0 | 0.00% | 0 | 0.00% | – |
| Combo (Chemo/ IO) | 0 | 0.00% | 0 | 0.00% | – | 0 | 0.00% | 0 | 0.00% | – | 1 | 2.38% | 1 | 2.63% | 0.942 | 0 | 0.00% | 0 | 0.00% | – |
| Combo (Chemo/ Target) | 0 | 0.00% | 0 | 0.00% | – | 0 | 0.00% | 1 | 0.99% | 0.273 | 0 | 0.00% | 0 | 0.00% | – | 0 | 0.00% | 0 | 0.00% | – |
| Combo (IO/ Hormone) | 1 | 0.29% | 0 | 0.00% | 0.354 | 0 | 0.00% | 0 | 0.00% | – | 0 | 0.00% | 0 | 0.00% | – | 0 | 0.00% | 0 | 0.00% | – |
| Combo (IO/Target) | 0 | 0.00% | 0 | 0.00% | – | 5 | 4.13% | 13 | 12.87% | 0.0175 | 0 | 0.00% | 0 | 0.00% | – | 0 | 0.00% | 0 | 0.00% | – |
| Combo (Chemo/ IO/Hormone) | 1 | 0.29% | 0 | 0.00% | 0.354 | 0 | 0.00% | 0 | 0.00% | – | 0 | 0.00% | 0 | 0.00% | – | 0 | 0.00% | 0 | 0.00% | – |
| Treatment Paradigm |  |  |  |  |  |  |  |  |  |  |  |  |  |  |  |  |  |  |  |  |
| Neoadjuvant | 5 | 1.47% | 3 | 1.03% | 0.619 | 0 | 0.00% | 0 | 0.00% | – | 6 | 14.29% | 3 | 7.89% | 0.366 | 0 | 0.00% | 0 | 0.00% | – |
| Adjuvant | 5 | 1.47% | 2 | 0.68% | 0.347 | 0 | 0.00% | 0 | 0.00% | – | 6 | 14.29% | 3 | 7.89% | 0.366 | 6 | 40.00% | 1 | 10.00% | 0.102 |
| Radical | 10 | 2.94% | 1 | 0.34% | 0.0128 | 0 | 0.00% | 0 | 0.00% | – | 1 | 2.38% | 0 | 0.00% | 0.338 | 7 | 46.67% | 0 | 0.00% | 0.0109 |
| Palliative | 320 | 94.12% | 284 | 97.26% | 0.0556 | 121 | 100.00% | 101 | 100.00% | – | 29 | 69.05% | 32 | 84.21% | 0.112 | 0 | 0.00% | 2 | 20.00% | 0.0710 |
| Curative | 0 | 0.00% | 2 | 0.68% | 0.126 | 0 | 0.00% | 0 | 0.00% | – | 0 | 0.00% | 0 | 0.00% | – | 2 | 13.33% | 7 | 70.00% | 0.00383* |
| Line of Palliative Treatment |  |  |  |  |  |  |  |  |  |  |  |  |  |  |  |  |  |  |  |  |
| 1 | 4 | 1.25% | 43 | 15.14% | <0.0001* | 72 | 59.50% | 57 | 56.44% | 0.644 | 18 | 62.07% | 15 | 46.88% | 0.234 | 0 | 0.00% | 2 | 100.00% | – |
| 2 | 213 | 66.56% | 216 | 76.06% | 0.0103 | 28 | 23.14% | 29 | 28.71% | 0.344 | 7 | 24.14% | 12 | 37.50% | 0.260 | 0 | 0.00% | 0 | 0.00% | – |
| 3 | 75 | 23.44% | 15 | 5.28% | <0.0001* | 9 | 7.44% | 11 | 10.89% | 0.371 | 3 | 10.34% | 4 | 12.50% | 0.792 | 0 | 0.00% | 0 | 0.00% | – |
| 4 | 19 | 5.94% | 6 | 2.11% | 0.0185 | 6 | 4.96% | 3 | 2.97% | 0.454 | 1 | 3.45% | 1 | 3.13% | 0.944 | 0 | 0.00% | 0 | 0.00% | – |
| 5 | 6 | 1.88% | 2 | 0.70% | 0.209 | 5 | 4.13% | 1 | 0.99% | 0.151 | 0 | 0.00% | 0 | 0.00% | – | 0 | 0.00% | 0 | 0.00% | – |
| 6 | 1 | 0.31% | 2 | 0.70% | 0.494 | 1 | 0.83% | 0 | 0.00% | 0.360 | 0 | 0.00% | 0 | 0.00% | – | 0 | 0.00% | 0 | 0.00% | – |
| 7 | 1 | 0.31% | 0 | 0.00% | 0.346 | 0 | 0.00% | 0 | 0.00% | – | 0 | 0.00% | 0 | 0.00% | – | 0 | 0.00% | 0 | 0.00% | – |
| Missing | 1 | 0.31% | 0 | 0.00% | 0.346 | 0 | 0.00% | 0 | 0.00% | – | 0 | 0.00% | 0 | 0.00% | – | 0 | 0.00% | 0 | 0.00% | – |
| Trial treatment |  |  |  |  |  |  |  |  |  |  |  |  |  |  |  |  |  |  |  |  |
| Yes | 50 | 14.71% | 41 | 14.04% | 0.812 | 7 | 5.79% | 4 | 3.96% | 0.533 | 7 | 16.67% | 7 | 18.42% | 0.837 | 0 | 0.00% | 0 | 0.00% | – |
| SACT initiated during study period |  |  |  |  |  |  |  |  |  |  |  |  |  |  |  |  |  |  |  |  |
| Yes | 74 | 21.76% | 57 | 19.52% | 0.488 | 22 | 18.18% | 21 | 20.79% | 0.624 | 14 | 33.33% | 17 | 44.74% | 0.296 | 8 | 53.33% | 7 | 70.00% | 0.405 |
| *Statistically significant *p*-values after Bonferroni correction for multiple comparisons | | | | | | | | | | | | | | | | | | | | |
